# Serial T2-Weighted Thoracic and Abdominal Lymphatic Imaging in Fontan Patients—New Insights into Dynamics of Lymphatic Abnormalities after Total Cavopulmonary Connection

**DOI:** 10.3390/jcdd9050138

**Published:** 2022-04-29

**Authors:** Julia Moosmann, Christian Schroeder, Oliver Rompel, Ariawan Purbojo, Sven Dittrich

**Affiliations:** 1 Department of Pediatric Cardiology, Friedrich-Alexander-Universität Erlangen-Nürnberg, 91054 Erlangen, Germany; christian.schroeder@extern.uk-erlangen.de (C.S.); sven.dittrich@uk-erlangen.de (S.D.); 2 Department of Radiology, Friedrich-Alexander-Universität Erlangen-Nürnberg, 91054 Erlangen, Germany; oliver.rompel@uk-erlangen.de; 3 Department of Pediatric Cardiac Surgery, Friedrich-Alexander-Universität Erlangen-Nürnberg, 91054 Erlangen, Germany; ariawan.purbojo@uk-erlangen.de

**Keywords:** Fontan, lymphatic abnormalities, T2-weighted imaging, magnetic resonance imaging

## Abstract

Lymphatic congestion in single-ventricle patients has been associated with increased morbidity and poor outcomes. Little is known about the dynamics of lymphatic abnormalities over time, on their association with clinical presentation or response to catheter interventions. This retrospective, single-center study describes Fontan patients who underwent at least two magnetic resonance imaging (MRI) studies. T2-weighted lymphatic imaging was used to classify thoracic and abdominal (para-aortic and portal-venous) lymphatic abnormalities. The relationship between lymphatic congestion and hemodynamic changes after cardiac catheter interventions, clinical presentation and MRI data was analyzed. A total of 33 Fontan patients underwent at least two cardiac MRI studies. Twenty-two patients had two, eight had three and three had four lymphatic imaging studies (total of 80 MRIs studies). No significant changes in lymphatic classification between MRI 1 and 2 were observed for thoracic (*p* = 0.400), para-aortic (0.670) and portal-venous (*p* = 0.822) abnormalities. No significant correlation between lymphatic classification and hemodynamic changes after intervention or MRI parameters was found. This study illustrates thoracic and abdominal lymphatic abnormalities in serial T2-weighted imaging after Fontan. Fontan patients did not demonstrate significant changes in their lymphatic perfusion, despite clinical or hemodynamic changes. We assume that lymphatic congestion might develop after total cavopulmonary connection (TCPC) and remain relatively stable, despite further intervention targeting hemodynamic parameters.

## 1. Introduction

Patients with single-ventricle physiology present with unique challenges associated with the Fontan circulation, which is characterized by passive pulmonary blood flow, decreased cardiac output, and increased central venous pressure (CVP) [1]. Chronic venous congestion in Fontan patients leads to alterations in lymphatic flow, contributing to the development of lymphatic abnormalities [2,3]. There is growing evidence that the degree of lymphatic congestion is associated with increased morbidity and the development of early complications and late Fontan failure [4,5,6].

Lymphatic abnormalities can be visualized with T2-weighted magnetic resonance imaging (MRI), which has become an important tool in the evaluation of the Fontan circulation and risk stratification [4,7,8]. Since the introduction of Biko et al.’s four-category classification, thoracic lymphatic abnormalities have been quantifiable in routine clinical practice [5]. There was an urgent need to extend this classification to the abdominal cavity for this patient population. Our group recently suggested a new classification for abdominal lymphatic abnormalities, including para-aortic and portal-venous lymphatic perfusion pattern [9]. This is important for two reasons: first, the lymphatic system is an interconnected network between the chest and abdominal cavity, and second, many patients present with abdominal symptoms, including ascites or protein-losing enteropathy (PLE) [10,11]. It is known that thoracic abnormalities may already be present after the Glenn procedure, which leads to an increased lymphatic afterload [5]. However, the dynamics in lymphatic congestion after the Fontan procedure are currently unknown. Furthermore, we do not know if we can influence the degree of lymphatic abnormalities with interventions targeting altered hemodynamics. This study aimed to evaluate whether lymphatic abnormalities after Fontan surgery change over time or are associated with changes in clinical findings.

## 2. Materials and Methods

### 2.1. Study Population

**Figure 1:**
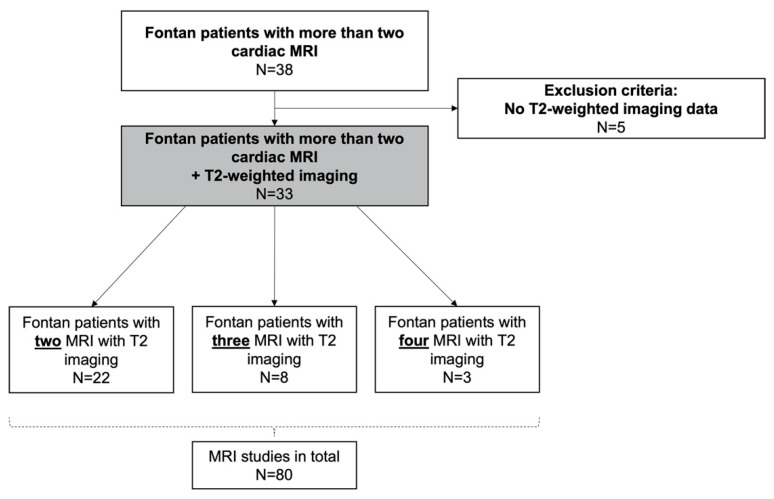
Flow diagram of study population.

The study population included patients who underwent the Fontan procedure between 2007 and 2020 and had at least two cardiac magnetic resonance imaging studies after total cavopulmonary connection (TCPC) at the Pediatric Heart Center of the University of Erlangen, Germany. Patients were identified by an in-house database search. Inclusion and exclusion criteria are illustrated in the flow chart (Figure 1).

### 2.2. Ethics Approval

This study was approved by the local ethic committee of the University of Erlangen Nürnberg (Ref.-No. 3738). Individual informed consent was waived for retrospective analysis of the data. The study was conducted in accordance with the Declaration of Helsinki.

### 2.3. Data Collection and Analysis

Demographics included sex, age at TCPC and underlying cardiac diagnosis. Cardiac MRI data were analyzed for ventricular end-diastolic volume (EDV in mL/m^2^), ventricular end-systolic volume (ESV in mL/m^2^), ventricular ejection fraction (EF%), and atrioventricular valve (AV) regurgitation. Data from cardiac catheterization included pressure values of superior vena cava (SVC) and transpulmonary gradient (TPG). Lymphatic abnormalities were evaluated with respect to hemodynamic changes after cardiac catheterization studies and correlated with SVC pressure and TPG. The degree of lymphatic congestion was correlated to cardiac MRI data (EF, EDV and ESV). Lymphatic congestion was analyzed depending on the indication of the study and whether early complications (as defined previously) occurred [4].

### 2.4. Magnetic Resonance Imaging Protocol after Total Cavopulmonary Connection

MRI was performed in the supine position under general anesthesia or spontaneous breathing (usually in patients older than 10 years). Scans were performed on a 1.5 tesla MR scanner equipped with high-performance gradients (Magnetom Aera, Siemens Healthineers, Erlangen, Germany). All studies imaged the neck, chest and abdomen. The imaging protocol included balanced steady-state free precession cine sequences for functional and volumetric analysis of both ventricles. The retrospectively gated ECG-triggered balanced steady-state free precession cine images were acquired during breath holding, covering the entire ventricular volume with a 10% gap.

Scan parameters were as follows for all patients: slice thickness 6 mm, in-plane resolution 2.5 mm × 1.8 mm, time to echo 1.2 ms, time to repetition 43 ms, and flip angle 50 degrees. Phased-contrast MRI was acquired for the quantification of valvular regurgitation fractions as well as flow velocities through valvular or vascular stenoses. Contrast-enhanced electrocardiography (ECG)-triggered MR angiography was conducted for three-dimensional visualization of ventricular morphology, cavopulmonary connections, pulmonary arteries and veins and the aortic arch. T2-weighted lymphatic imaging was performed using BLADE, a motion insensitive, multi-shot Turbo Spin Echo sequence with fat saturation. This technique acquires images during breath holding; an intershot motion correction is applied for reducing image artifacts due to pulsation, intestinal peristalsis, or residual respiratory movement. Scan parameters were as follows: total measurement time approximately 3 min, coronal orientation with complete coverage of the neck, chest and abdomen, slice thickness 6 mm and slice gap of 1.5 mm, in-plane resolution 1.1 × 1.1 mm^2^, time to echo 110 ms, time to repetition 1800 ms and flip angle 160 degrees [9].

### 2.5. MRI Analysis

Image data analysis was performed using syngo.via software (Siemens Healthineers, Erlangen, Germany) enabling post-processing of cardiac MRI data. EDV and ESV were calculated by summarizing the volume of the ventricular blood pool in each section. EF was calculated based on EDV and ESV [EF  =  (end-diastolic volume − end-systolic volume)/end-diastolic volume] × 100 (%). T2-weighted images were analyzed with the same software. All measurements were performed as previously described [9].

### 2.6. T2-Weighted Imaging and Classification of Lymphatic Abnormalities

T2-weighted MRI sequences illustrate lymphatic abnormalities as areas of higher intensity [4,5]. Description of lymphatic abnormalities was performed under consideration of anatomical landmarks to avoid interference with other high-intensity structures. Thoracic and abdominal lymphatic abnormalities were classified by two experienced pediatric cardiologists blinded to history and outcome of the patients.

Thoracic lymphatic abnormalities were graded according to the classification of Biko et al. [5]:

- Type 1: little or no presumed lymphatic channels within the supraclavicular region and mediastinum;
- Type 2: abnormal increased lymphatic channels within the supraclavicular region without extension into the mediastinum;
- Type 3: abnormal supraclavicular lymphatics with extension into the mediastinum;
- Type 4: abnormal supraclavicular lymphatic channels with extension both into the mediastinum and in the interstitial pattern into the lung parenchyma.

Abdominal lymphatic abnormalities were graded according to the recently described classification [9].

1. Para-aortic lymphatic abnormalities:
- Type 1: little or no para-aortic lymphatic abnormalities;
- Type 2: para-aortic lymphatic abnormalities not following the renal arteries;
- Type 3: para-aortic lymphatic abnormalities following the renal arteries;
- Type 4: para-aortic lymphatic abnormalities following the renal arteries with extension to the inguinal region.
2. Portal-venous lymphatic abnormalities:
- Type 1: little or no portal-venous lymphatic abnormalities;
- Type 2: portal-venous lymphatic abnormalities not following the mesenteric veins;
- Type 3: portal-venous lymphatic abnormalities following the mesenteric veins.

### 2.7. Statistics

Demographic, clinical, operative and outcome variables were reported using descriptive statistics and are expressed as median and interquartile range (IQR). Mann–Whitney-U test was used to compare ordinal data between two groups (e.g., lymphatic classification MRI 1 and 2). Kruskal–Wallis test was used for group comparisons (e.g., to identify changes for patients with more than 3 MRIs). Correlations were calculated using Spearman correlation. All statistical analyses were performed using IBM SPSS statistics v24.0 and GraphPad Prism (version 9.3.1).

## 3. Results

### 3.1. Patient Demographics and History

A total of 33 patients were identified who had at least two cardiac MRI studies with T2-weighted imaging (Figure 1). Demographic details of patients are illustrated in Table 1. Median time between TCPC and first cardiac MRI was 7.5 (IQR 33) months, the second MRI 4.5 (IQR 3.6) years later. In total, 80 cardiac MRIs with T2-weighted imaging were analyzed.

### 3.2. Changes in Lymphatic Abnormalities after Total Cavopulmonary Connection

**Figure 2:**
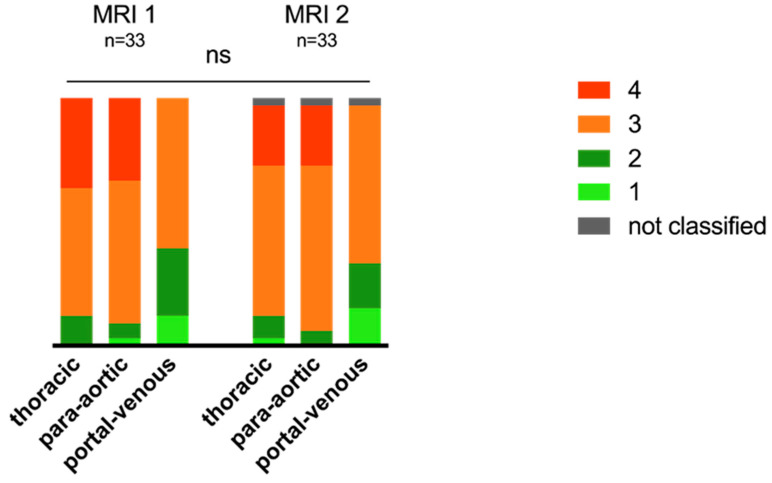
Changes in classification for thoracic and abdominal lymphatic congestion in MRI 1 and 2. Stacked bar graph illustrating distribution of lymphatic classification: light green type 1, dark green type 2, orange type 3, red type 4 and grey not classified. Thirty-three patients underwent two MRIs. There were no significant changes in lymphatic classification between MRI 1 and 2 (not significant = ns).

Based on the imaging classification noted above, the distribution of thoracic and abdominal lymphatic abnormalities at MRI 1 and 2 is illustrated in Figure 2. No significant changes in lymphatic classification between MRI 1 and 2 for thoracic (*p* = 0.400), para-aortic (*p* = 0.670) and portal-venous (*p* = 0.822) abnormalities were observed. There were no significant changes for patients who underwent three MRIs in the thoracic (*p* = 0.798), para-aortic (*p* = 0.670) or portal-venous (*p* = 0.822) classification.

Between MRI 1 and 2, nine (27.3%) patients did not show any changes in their thoracic, para-aortic or portal-venous lymphatic abnormalities, while 24 (72.7%) patients showed changes in their classification. Despite changes in classification being observed in most patients, those were mostly mild and, therefore, did not meet statistical significance. Clinically, it has been shown that higher-grade (type 3 and 4) abnormalities show reduced outcome [5]. In total, four (12.1%) patients changed from low-grade (type 1 or 2) congestion to higher-grade, and four (12.1%) patients from higher-grade abnormalities to a lower classification.

An improvement in lymphatic abnormalities was observed in 11 (45.8%) patients and a deterioration in nine (37.5%). Four (16.7%) patients showed variation in their lymphatic classifications, including an improvement in one of the three classifications and a deterioration in one or two of the other classifications. Improvement in the classification by two points was observed in two (6.1%) patients (one improved in the portal-venous classification and one in the para-aortic classification). Both patients presented with prolonged pleural effusions after TCPC for more than four weeks. One patient underwent surgical ligation of lymphatic fistulae to the pleura and subsequently improved clinically and on the following T2-weighted imaging. The second patient showed prolonged effusions after TCPC, which resolved following increased diuretic therapy and a low-fat diet. Two (6.1%) patients showed a deterioration by two points in the para-aortic and portal-venous classification. Both patients did not show new clinical symptoms or signs of failure. All other patients showed mild changes through variation in one in their classification (18 patients (54.5%)).

Thoracic lymphatic classification varied in 15 (45.5%) patients, with nine (60%) patients demonstrating a mild improvement and six (40%) a deterioration in their lymphatic classification. Eleven (33.3%) patients presented with changes in their para-aortic classification, with seven (63.6%) improving and four patients (36.4%) worsening. The portal-venous classification changed in eleven (33.3%), with five (45.5%) patients improving and six (54.5%) worsening (Figure 2). Individual changes are illustrated in Figure S1. An improvement in the thoracic lymphatic classifications was associated with an improvement in another compartment in 33%. Improvement in para-aortic lymphatic congestion was associated with an improvement in thoracic or portal-venous congestion in 57% and an improvement in portal-venous congestion was associated with improvements in the other classification in 66%.

Clinical indication for follow-up MRI and respective changes in lymph classification are illustrated in Table 2. Early complications were identified in 12 (36%) patients. The association between lymphatic classifications at MRI 1 and 2 and early complications are illustrated in Table 3.

### 3.3. Influence of Hemodynamics and Catheter Interventions on Lymphatic Abnormalities

No correlation was observed between EF (%) and the degree of thoracic (*p* = 0.644), para-aortic (*p* = 0.886) or portal-venous (*p* = 0.650) lymphatic abnormalities at MRI 1. No correlation was found between EF and thoracic (*p* = 0.472), para-aortic (*p* = 0.063) and portal-venous (*p* = 0.198) at MRI 2, and there was no significant correlation between EDV and thoracic or portal-venous lymphatic abnormalities. EDV correlated significantly with para-aortic congestion (*p* = 0.015) at MRI 2 but not MRI 1 (*p* = 0.642) (see Table 4).

**Figure 3:**
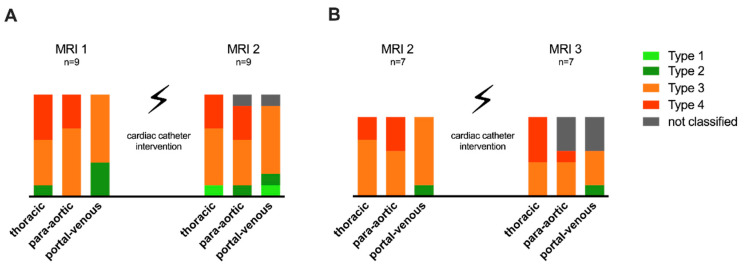
Changes in lymphatic abnormalities after hemodynamic intervention. (**A**) Changes in lymphatic abnormality classification between MRI 1 and 2: including nine patients. (**B**) Changes between MRI 2 and 3: including seven patients.

**Figure 4:**
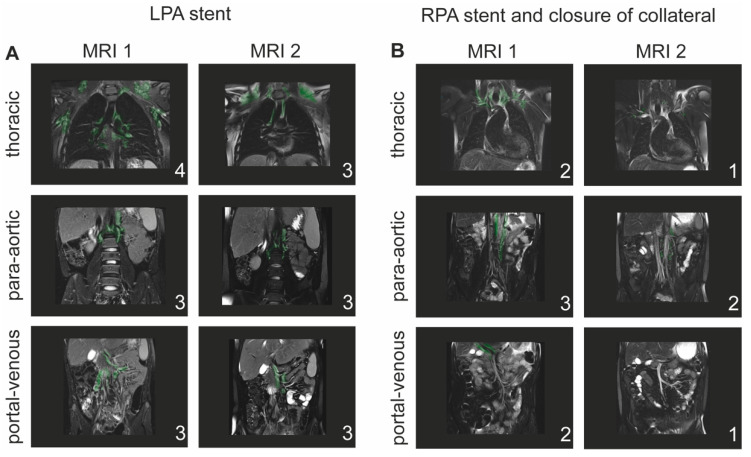
Example of lymphatic abnormalities after cardiac catheter intervention. (**A**) Patient who underwent first cardiac MRI at 13 years of age showing moderate left pulmonary artery (LPA) stenosis. The T2-weighted imaging showed significant thoracic lymphatic abnormalities (type 4) and para-aortic and portal-venous abnormalities type 3. The 2nd MRI was performed after cardiac catheterization and LPA stent implantation showing mildly improved thoracic lymphatic abnormalities. (**B**) 25-year-old Fontan patient in good clinical condition presented with significant stenosis of the right pulmonary artery (RPA) on 1st MRI. Cardiac catheter intervention with balloon dilatation of the RPA and closure of a major aortopulmonary collateral (MAPCA) from the descending aorta was performed. Repeat MRI at the age of 30 years showed improvement in lymphatic abnormalities in all three compartments. Lymphatic abnormalities are highlighted in green.

Ten patients required one or more cardiac catheter studies between MRI 1 and 2 and seven patients between MRI 2 and 3. Changes in the lymphatic classification for patients requiring catheter intervention are illustrated in Figure 3. An example of T2-weighted imaging pre- and post-catheter intervention, with improved lymphatic abnormalities after cardiac catheter interventions, is demonstrated in Figure 4. No correlation between Fontan hemodynamics (SVC pressure and TPG) with the degree of lymphatic abnormalities on the following T2-weighted imaging study was found (Table 5).

## 4. Discussion

This study illustrates the dynamics of thoracic and abdominal lymphatic abnormalities during follow-up after TCPC with T2-weighted imaging. Overall, we identified that lymphatic abnormalities remain relatively stable, despite interventions aiming to improve hemodynamics and changes in clinical presentation.

Lymphatic congestion in univentricular patients has been associated with early complications and late Fontan failure [4,5]. Therefore, the illustration of lymphatic abnormalities with T2-weighted imaging is increasingly recognized as an important diagnostic tool for risk stratification, before and after TCPC [4,7]. The development of lymphatic abnormalities has been associated with chronic venous stasis, secondary to increased central venous pressure (CVP) and increased lymph production, leading to lymphatic congestion in the univentricular circulation. A classification for thoracic lymphatic abnormalities was described by Biko et al., and recently, abdominal lymphatic abnormalities have been classified by our group for improved clinical quantification [5,9]. The abdominal lymphatic perfusion pattern is of particular interest after TCPC due to the role of the liver, which produces about 25–50% of the lymph flow in the thoracic duct. Hepatic lymph production can be even further accelerated in the case of hepatic congestion and liver fibrosis, which plays a role in the Fontan circulation [10,12,13]. Despite the newly introduced classifications, little is known about the dynamics of lymphatic abnormalities during follow-up and in relation to clinical changes.

The majority of patients in our cohort underwent T2-weighted imaging shortly after TCPC and the second MRI 4.5 years later. Overall, we did not observe significant changes in lymphatic abnormalities and most alterations were a change by one in the lymph classification or a mild variation. Specifically, higher-grade lymphatic malformations have been associated with poor outcome after Fontan [5]. We did not observe significant shifts from high-grade abnormalities to lower classifications.

After the superior cavopulmonary connection (SCPC), patients present with increased afterload on lymphatic drainage and mild lymphatic congestion but usually in the context of normal lymphatic production. Therefore, most patients do not develop significant lymphatic perfusion abnormalities after the Glenn procedure.

**Figure 5:**
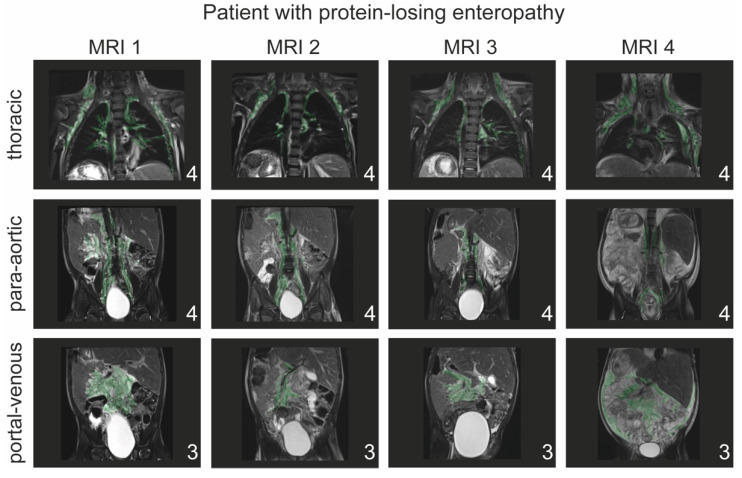
Example of patient with protein-losing enteropathy. Patient who presented with prolonged pleural effusion (28 days) and edema after TCPC requiring diuretic therapy. Over the next two years, the patient presented with continuous clinical deterioration requiring frequent admissions to hospital for volume overload, recurrent pleural effusion, diarrhea and protein loss. The patient underwent serial cardiac MRI and cardiac catheter studies targeting hemodynamic parameters. Despite clinical variation in the presentation, lymphatic classification did not change during the entire period. A lymphatic fistula to the left lower lobe bronchus and thoracic cavity can be visualized at MRI 1 and remains visible at all four MRIs. At the time of MRI 4 the patient presented with clinical deterioration and a pleural effusion on the left side and ascites in addition to the same degree of lymphatic abnormalities. Lymphatic abnormalities are highlighted in green.

After Fontan, CVP increases and there is increased preload on the liver. This, in addition to high lymphatic afterload, reduces the lymphatic drainage capacity. However, not all patients with a Fontan circulation develop severe lymphatic abnormalities and it is hypothesized that the physiologic changes in venous and lymphatic congestion, plus an anatomical variant of the lymphatic vasculature, might lead to lymphatic failure in some of those patients [14]. This would explain why some patients with high CVP do not develop lymphatic failure and some who seem to have favorable hemodynamics might show severe lymphatic dysfunction [2,3,15,16]. In our cohort, we did not identify any correlation between ventricular dysfunction or hemodynamic parameters with lymphatic classification. Catheter interventions aiming to improve hemodynamics in the univentricular circulation remain important, particularly in patients with signs of failure, and might be beneficial for CVP and, therefore, the lymphatic afterload. However, it might influence present lymphatic abnormalities only mildly (Figure 5).

We hypothesized that if lymphatic abnormalities develop, they persist despite clinical changes [17], unless they underwent lymphatic intervention, as illustrated in one case of our cohort. Once the lymphatic vessels are dilated and filled with lymph fluid, they remain visible in T2-weighted imaging, with some variation, depending on current lymph production and fluid status.

About 33% of patients in our cohort experienced early complication, similar to the numbers reported in the current literature [5]. An example of serial T2-weighted imaging in one patient with prolonged pleural effusion after TCPC who developed PLE protein-losing enteropathy two years after surgery is illustrated in Figure 5.

T2-weighted imaging has become increasingly important for single-ventricle patients and represents a ‘game changer’ to screen patients with Fontan failure, including PLE and plastic bronchitis. However, new modalities have been described to illustrate the lymphatic perfusion, including nodal, hepatic and mesenteric dynamic contrast lymphangiography (DCMRL) [11,16,18]. DCMRL allows for a full characterization of lymphatic perfusion, including physiology, anatomy, and flow of the lymphatic system. Patients with significant clinical problems and higher-grade lymphatic abnormalities in (serial) T2-weighted imaging might benefit from additional DCMRL to understand the etiology and gain information for interventional planning. However, DCMRL is not yet performed routinely in Fontan patients with symptoms of failure and might not be available at all centers.

### Limitations

This is a retrospective study of a small cohort of selected patients who underwent serial T2-weighted imaging. One major limitation is a potential selection bias for the follow-up MRI. A prospective approach to evaluate lymphatic abnormalities at definite time points is needed in the future.

## 5. Conclusions

Analysis of serial T2-weighted imaging during follow-up after TCPC has demonstrated that lymphatic congestion, once developed after TCPC, remains mostly stable, and changes in classification of thoracic or abdominal abnormalities underly only limited variation.

## Figures and Tables

**Table 1:** Demographics and clinical characteristics of study cohort.

Demographics	
Sex	
Male (*n*)	19 (57.6%)
Female (*n*)	14 (42.4%)
Age at milestones	
Age at first surgery (days)	9 (15)
Age at Glenn prodecure (months)	6 (8)
Age TCPC (years)	3.3 (9)
Age 1st MRI (years)	4 (6)
Time between TCPC and 1st MRI (months)	7.5 (33)
Age at 2nd MRI (years)	10 (8)
Age at 3rd MRI (years) *n* = 11	10 (9)
Age at 4th MRI (years) *n* = 3 Age at last follow-up (years)	11 (6) 13.5 (8)
Systemic left ventricle (*n*)	15 (45.4%)
Systemic left ventricle (*n*)	16 (48.5%)
Single ventricle with both components (*n*)	2 (6.1%)
**Fenestration (*n*)**	2 (6.1%)
**Conduit size (mm)**	18 (2)
**Syndromes**	
Trisomy 21	3 (9.1%)
DiGeorge syndrome (22q11.2)	1 (3.0%)
**Cardiac malformations (*n*)**	
Hypoplastic left heart syndrome	6 (18.2%)
Unbalanced atrioventricular septal defect	4 (12.1%)
Double-inlet left ventricle	4 (12.1%)
Double-outlet left ventricle	4 (12.1%)
Pulmonary atresia with intact ventricular septum	3 (9.1%)
Hypoplastic or interrupted aortic arch with LV hypoplasia	3 (9.1%)
Tricuspid atresia	6 (18.2%)
Congenitally corrected transposition of great arteries	2 (6.1%)
Criss-cross heart	1 (3.0%)
Early complications (*n*)	12 (36.4%)
Death (*n*)	5 (15.2%)
**Death causes and age at death (years)**	
Postoperative death after conduit change	9
Portal vein thrombosis	15
Cerebral haemorrhage	9
Thromboembolic event	7
Acute transplant rejection	23

Data are given as number (percentage) or median and interquartile range (IQR). Abbreviations: Total cavopulmonary connection (TCPC), magnetic resonance imaging (MRI), left ventricular (LV).

**Table 2:** Indication for follow-up MRI and clinical changes in lymphatic abnormalities.

Indication for 2nd MRI	Changes in Lymph Classification	Numbers of Patients (*n*)
**Clinical indication** (*n* = 12, 36.6%)		
Symptoms of protein-losing enteropathy, new onset or worsening of oedema, pleural effusion, or ascites (*n* = 7, 21.2%)	Stable	1 (14.3%)
	Improved	3 (42.9%)
	Worsening	2 (28.6%)
	Variation	1 (14.3%)
Cyanosis and reduced exercise capacity (*n* = 4, 12.2%)	Stable	3 (75.0%)
	Variation	1 (25.0%)
SVT (*n* = 1, 3%)	Variation	1 (100%)
**Morphologic/hemodynamic question** (*n* = 2, 6.1%)	Stable	1 (50.0%)
	Variation	1 (50.0%)
**Regular follow-up** (*n* = 19, 57.6%)	Stable	4 (21.1%)
	Improved	6 (31.6%)
	Worsening	6 (31.6%)
	Variation	2 (10.5%)

Data are given as number (percentage). Abbreviations: supraventricular tachycardia (SVT), magnetic resonance imaging (MRI).

**Table 3:** Early complications and lymphatic abnormalities.

	Lymphatic Classification	Total *n* = 33	Early Complication *n* = 12	No Complication *n* = 21
MRI 1	**Thoracic**			
	Type 1	-	-	-
	Type 2	4	-	4 (19%)
	Type 3	17	6 (50%)	11 (52%)
	Type 4	12	6 (50%)	6 (28%)
	**Para-aortic**			
	Type 1	1	1 (8%)	-
	Type 2	2	-	2 (10%)
	Type 3	19	5 (42%)	14 (67%)
	Type 4	10	6 (50%)	5 (23%)
	**Portal-venous**			
	Type 1	2	1 (8%)	3 (14%)
	Type 2	9	3 (24%)	6 (28%)
	Type 3	20	8 (68%)	12 (57%)
MRI 2	**Thoracic**			
	Type 1	1	-	1 (5%)
	Type 2	3	-	3 (14%)
	Type 3	19	7 (58%)	12 (57%)
	Type 4	9	4 (41%)	4 (19%)
	**Para-aortic**			
	Type 1	-	-	-
	Type 2	2	1 (8%)	1 (5%)
	Type 3	23	8 (68%)	16 (76%)
	Type 4	7	3 (24%)	4 (19%)
	**Portal-venous**			
	Type 1	5	2 (16%)	3 (14%)
	Type 2	6	1 (8%)	6 (28%)
	Type 3	21	9 (75%)	12 (57%)

Data are given as number (percentage).

**Table 4:** MRI parameters and lymphatic abnormalities.

	1st MRI	2nd MRI	3rd MRI	4th MRI
	*n* = 33	*n* = 33	*n* = 11	*n* = 3
**Indication for MRI**				
Routine/follow-up	26 (78.8%)	19 (57.6%)	6 (54.5%)	3 (100%)
Clinical indication	6 (18.2%)	12 (36.4%)	5 (45.5%)	
Morphologic question	1 (3.0%)	2 (6.1%)		
**Body weight (kg)**	16.8 (9.2)	37 (21.1)	31 (23.8)	39.6 (16.6)
**End-diastolic volume (mL/m^2^ BSA)**	76.5 (32.6)	79 (21)	77 (19)	68 (13.5)
**End-systolic volume (mL/m^2^ BSA)**	35.5 (23)	39.5 (21)	40 (9)	31 (4.7)
**Ventricular ejection fraction (%)**	50.5 (11)	49.5 (17)	53 (13)	56 (5.5)
**Relevant AV-valve regurgitation (*n*)**				
Mild	10 (30.3%)	13 (39.4%)	3 (9.1%)	3 (9.1%)
Moderate	2 (6.1%)	4 (12.1%)		
Severe		1 (3.0%)		
**Oxygen saturation (%)**	95 (3)	95 (4.8)	95 (2.5)	97 (3)

Data are given as number (percentage) or median and interquartile range (IQR). Abbreviations: atrioventricular (AV), body-surface area (BSA), magnetic resonance imaging (MRI).

**Table 5:** Correlation of cardiac catheterization data and lymphatic abnormalities.

Timing of Cardiac Catheterization and Hemodynamic Assessment	Patients (*n*)	Pressure (mmHg)	Lymphatic Abnormalities		
			Thoracic	Para-Aortic	Portal-Venous
**Cardiac catheterization pre-Fontan**					
TPG	27 (81.8%)	4.5 (2.75)	0.318 *	0.425 *	0.706 *
SVC		11 (3)	0.909 *	0.273 *	0.532 *
**Cardiac catheterization between TCPC and MRI 1**	11				
SVC	(33.3%)	16 (3.5)	0.626 *	0.149 *	0.435 *
**Cardiac catheterization between MRI 1 and 2**					
SVC	10	12 (4)	0.994 #	0.097 #	0.056 #
TPG	(30.0%)	5 (3)	0.242 #	0.587 #	0.999 #
**Cardiac catheterization between MRI 2 and 3**	7				
SVC	(21.2%)	14 (3.5)	0.999 **	0.500 **	0.500 **

Table illustrating pressure values at cardiac catheter studies. The pressure values have been correlated with the lymphatic classification of the following MRI: * with results from 1st MRI. **#** with results from 2nd MRI. ** with results from 3rd MRI. Data are given as number (percentage) or median and interquartile range (IQR). Abbreviations: transpulmonary gradient (TPG), superior vena cava (SVC), magnetic resonance imaging (MRI), total cavopulmonary connection (TCPC).

## Data Availability

The data presented in this study are available on request from the corresponding author. The data are not publicly available due to the potential for identifying patient information (e.g., MRI dataset).

## Supplementary Materials

The following supporting information can be downloaded at: https://www.mdpi.com/article/10.3390/jcdd9050138/s1, Figure S1: Individual changes in lymphatic abnormalities. Heat map illustrates individual changes of classifications: light green type 1, dark green type 2, orange type 3, red type 4 and grey not classified. *n* = 33 patients underwent two MRIs. *n* = 11 patients underwent three MRIs and *n* = 3 patients underwent four follow-up imaging studies.
