# Supplementary material for: Serial T2-Weighted Thoracic and Abdominal Lymphatic Imaging in Fontan Patients—New Insights into Dynamics of Lymphatic Abnormalities after Total Cavopulmonary Connection
Source: J Cardiovasc Dev Dis. 2022 Apr 29;9(5):138. doi: 10.3390/jcdd9050138 (PMC9144783; doi:10.3390/jcdd9050138)
Supplement: Supplementary file 1 [file jcdd-09-00138-s001.zip › jcdd-1670937-supplementary.pdf]

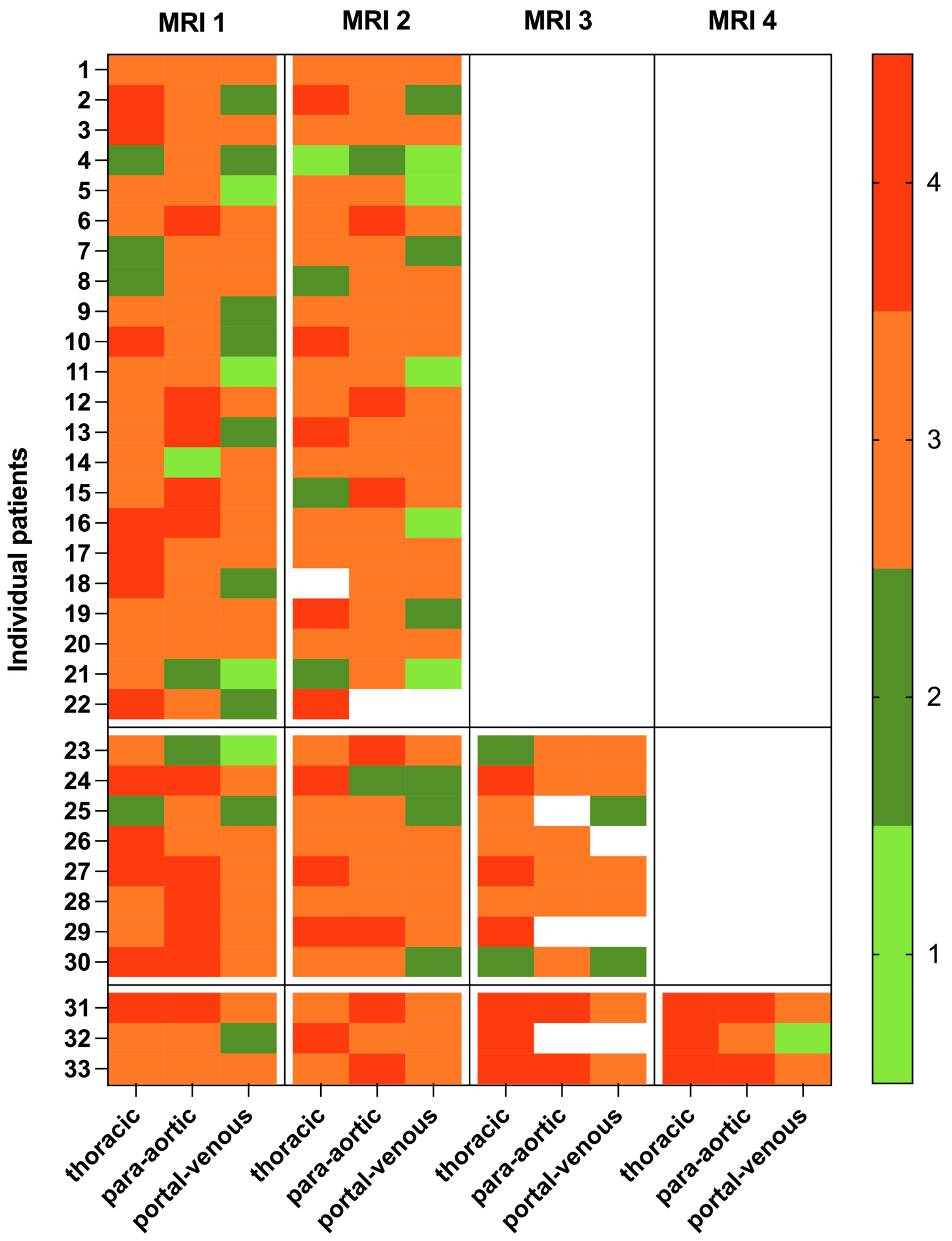


**Figure S1.** Individual changes in lymphatic abnormalities. Heat map illustrates individual changes of classifications: light green type 1, dark green type 2, orange type 3, red type 4 and grey not classified. N = 33 patients underwent two MRIs. N = 11 patients underwent three MRIs and N = 3 patients underwent four follow-up imaging studies.
